# Cumulative Effect of Psychosis and Aging on Cognitive Function in Patients Diagnosed With Schizophrenia Spectrum Disorders: A Cognitive Domain Approach

**DOI:** 10.7759/cureus.66733

**Published:** 2024-08-12

**Authors:** Panagiotis A Malliaris, Nikiforos V Angelopoulos, Efthimios Dardiotis, Konstantinos Bonotis

**Affiliations:** 1 Department of Psychiatry, Athens General Hospital "Evangelismos", Athens, GRC; 2 Department of Psychiatry, Faculty of Medicine, School of Health Sciences, University of Thessaly, Larissa, GRC; 3 Department of Neurology, Faculty of Medicine, School of Health Sciences, University of Thessaly, Larissa, GRC

**Keywords:** cognitive assessment, schizophrenia spectrum disorders, cognitive decline, psychosis, aging

## Abstract

Background: Schizophrenia spectrum disorders are characterized by cognitive decline, which is evident even in the prodromal phase. Aging is a complex gradual procedure that affects, among other organs, the central nervous system, resulting in age-related cognitive decline.

Objective: The objective of this study is to assess the cognitive function of patients diagnosed with psychotic disorders, in comparison with healthy controls, along the age spectrum.

Methods: Sixty patients diagnosed with schizophrenia spectrum disorders in remission, 20-59 years old, and 60 healthy controls, matched by age and educational level, from the region of Thessaly in Central Greece, were evaluated, with respect to their cognitive performance, using the Greek version of the Montreal Cognitive Assessment (MoCA). Correlations between age and MoCA total and cognitive domains’ scores, as well as statistical analysis of variance (ANOVA) and t-test among age groups, were performed using Statistical Product and Service Solutions (SPSS, version 23; IBM SPSS Statistics for Windows, Armonk, NY).

Results: The MoCA score was negatively correlated with age, both in the patients’ group (p<0.001) and in the control group (p=0.001). A significant statistical difference in mean MoCA scores between patients and healthy controls was observed, not only in the total sample (p<0.001) but also in all age groups (20-29: p=0.006, 40-49: p=0.024, 50-59: p<0.001), except for age group 30-39 (30-39: p=0.356). Statistically significant differences were also found between patients and healthy controls in the total sample, regarding specific cognitive domains, in the visuospatial and executive function domain (p=0.01), attention domain (p<0.001), language domain (p<0.001), and orientation domain (p<0.005). Interestingly, different deterioration patterns in cognitive domains were observed in each age group. Specifically, in the age group 20-29, statistically significant differences were found between patients and healthy controls in the language domain (p<0.014) and orientation domain (p<0.041). No difference was found in the age group 30-39, while statistically significant differences were found between patients and healthy controls in the age group 40-49 in the attention domain (p<0.001) and language domain (p<0.001). Finally, in the age group 50-59, such differences were found in the visuospatial and executive function domain (p=0.041), attention domain (p=0.006), and language domain (p=0.001). Statistically significant cognitive decline occurs in a shorter period in the patients’ group, suggesting an accelerated cognitive decline in psychotic patients after middle age.

Conclusions: Age-related cognitive decline in psychotic patients occurs at an accelerated rate in relation to the control sample, with age-specific cognitive domain decline patterns, due to the cumulative effect of aging and psychosis on cognition. Further, larger, multicenter research should focus on establishing these results and designing relevant procognitive interventions.

## Introduction

Psychosis-related cognitive decline

Schizophrenia is the main diagnostic representative of the schizophrenia spectrum and other psychotic disorders, as described in the Diagnostic and Statistical Manual of Mental Disorders, Fifth Edition (DSM-5) [1]. Psychosis is a wider clinical term, describing a condition of thought and behavioral dysfunction, related to disorders of perception of reality and others, which is apparent in many psychiatric, neurodevelopmental, neurological, and medical conditions [2]. Although schizophrenia represents approximately 30% of the cases of the much larger multidimensional spectrum of psychotic disorders, it attracts the largest part of scientific research efforts at the genetic, molecular, and neuronal levels, but due to its heterogeneity, the search for special biomarkers or etiopathogenetic subcategories is still unsuccessful [3].

The conceptualization of psychotic disorders as a spectrum implies that a dimensional approach might be more suitable. Therefore, an eight-dimensional model is suggested in DSM 5, according to which eight symptoms are assessed in parallel with the use of the Clinician-Rated Dimensions of Psychosis Symptom Severity (CRDPSS) measure [1]. This approach not only incorporates mood disorders with psychotic features, such as bipolar disorder, but also confirms the consideration of cognitive decline as a core symptom of the schizophrenia spectrum and other psychotic disorders [4].

Psychotic disorders and early-onset cognitive decline were first strongly connected, since Kraepelin had originally named the syndrome “dementia praecox”, focusing on the cognitive deficits seen in the patients. Despite the predominance of the term schizophrenia in the following years, the effects of psychosis on cognitive function have been well established, in the form of structural and functional changes in the central nervous system [5]. Volumetric studies have shown a significant reduction in grey matter volume, localized in specific brain areas, such as the anterior cingulate and insula, suggesting a disorder-specific pattern of structural brain reorganization [6], resulting in less efficient neural networks, due to abnormal temporal synchronization [7]. It is noteworthy that the level of heritability of the above-mentioned cognitive-related structural and functional alterations is increased in schizophrenia [8].

Recent research efforts focusing on cognitive decline in schizophrenia spectrum disorders point out two discrete mechanisms of cognitive deterioration, an acute phase during psychotic episodes that regresses partially after remission [9], and a long-term, neurodevelopmental procedure that begins before birth and is apparent even in the prodromal phase [10], as a result of multiple genes susceptible for risk of schizophrenia’s heritability, as shown by genome-wide association studies [11]. Some of these 108 genes are related to glutaminergic neuronal pathways, neuroplasticity, and neurodevelopmental procedures and affect working memory, verbal memory, and concentration [12]. Thus, malfunctioning genes related to perception and data processing may induce hallucinations and delusions, respectively [13], and susceptible genes related to executive functions may lead to negative symptoms [14]. In addition to genetic susceptibility, multiple modifiable environmental factors, such as medical comorbidities (e.g., hypertension, obesity, and diabetes mellitus), head injury, air pollution, smoking, psychiatric comorbidities (e.g., depression and alcohol consumption), exercise, education, social interaction, and hearing loss, affect cognition throughout lifetime [15].

When genetic susceptibility is combined with environmental factors, neurodevelopmental dysfunctions, and epigenetic alterations, brain connectivity distortion and integration dysregulation between sensory systems and higher processing centers occur [16]. During the prodromal phase of psychotic disorders, an increased frontoparietal cognitive effort is needed to maintain intact reality testing, but when it finally decompensates, these above-mentioned procedures surpass the clinical threshold, resulting in psychotic symptoms, such as delusions and hallucinations [17]. Consequently, distorted cognition is not only a symptom but affects directly and indirectly the etiopathology and symptomatology of schizophrenia spectrum disorders.

The cognitive function of psychotic patients, during disorders, is also affected by neurodegenerative factors, such as a chronic inflammatory process in the central nervous system, mediated by proinflammatory cytokines and microglia abnormalities [18]. Furthermore, the initiation of antipsychotic medication after the manifestation of psychotic symptoms and the consequent diagnosis has a complex result in cognition [19]. Controlling positive symptoms contributes to the remission of acute cognitive deterioration seen during psychotic episodes [9], but the metabolic and cardiovascular side effects of novel antipsychotics have chronic implications and are linked to cognitive decline [19]. Consideration of other environmental and social factors in the cognitive function of psychotic patients, such as consequences of nonadherence to medication, absence of insight, and lack of adequate social support and reduced social interactions, further highlights the complexity of the phenomenon.

Age-related cognitive decline

Molecular Mechanisms of Age-Related Cognitive Decline

Aging is a complex gradual procedure characterized by a delayed and progressive accumulation of degenerative molecular and cellular damage [20]. Oxidative stress and the production of reactive oxygen species from cellular metabolism, due to mitochondrial dysfunction and the antioxidant system’s dysregulation, damage structural and functional proteins, as well as nucleic acids [20]. Therefore, epigenetic DNA methylation and histone acetylation, as well as accumulation of advanced glycation end products (AGEs) occur, accompanied by cellular senescence and telomere shortening [20,21]. The gradual accumulation of molecular and cellular damage manifests itself as clinical deterioration in system and organ functionality, among which vascular damage is crucial, especially in the central nervous system, which is very sensitive to ischemia [22]. This ubiquitous phenomenon contributes to age-related cognitive decline and is related to the gradually reduced neuroplasticity of the brain, mediated by the dysregulation of sirtuins and brain-derived neurotrophic factors [23].

Furthermore, in terms of brain functionality, which is characterized by increased metabolic activity, mitochondrial dysregulation plays an important role in age-related cognitive decline [21]. In addition, other specific changes in the central nervous system, such as the gradual decrease in neurogenesis rate, increased the number of oligodendrocytes and astrocytes, myelin dysfunction, increased microglial activity, and consequent chronic inflammation [23], contribute to age-related cognitive decline. These changes lead to the accumulation of β-amyloid and the formation of senile or amyloid plaques and neurofibrillary tangles, polymers of TAU protein, due to proteostasis dysfunction [24]. When additional genetic or environmental factors co-exist, this effect is magnified and neurodegenerative disorders are diagnosed, such as Alzheimer’s disease [24].

Early Psychological Life Events and Later Cognitive Decline

The abovementioned molecular events mediate neurodegenerative mechanisms that are accumulated during the lifespan and result in age-related cognitive decline. These procedures are not only determined by genetic susceptibility but are also influenced by environmental factors that can induce temporary, as well as permanent effects, due to epigenetic alterations. A crucial factor that affects cognitive functionality is the impact of early psychological life events. Early life adversity can induce inflammation and influence the gut microbiome, the hypothalamic-pituitary-adrenal axis, the myelination of oligodendrocytes, and the adult neurogenesis in the hippocampus, resulting in increased vulnerability for cognitive impairment later in life [25].

Cognitive Domain-Specific Age-Related Cognitive Decline

Age-related cognitive decline affects a variety of cognitive functions, resulting in slower data processing, impaired executive function, and memory disorders [24]. Other age-related factors that affect cognitive functions are circadian dysregulation and alterations in sleep patterns, as well as increased vulnerability to depression [20]. Successful executive functioning depends on brain connectivity and adequate integration of neuronal centers. Disconnected aging is an age-related phenomenon characterized by decreased brain connectivity, due to dysfunction in neuroplasticity and accumulation of irreparable degenerative structural damage [26]. This procedure results in slower data processing and disorders in executive functions, such as attention focus distortion, difficulty in the execution of complex tasks, and working memory disorders, which are apparent in normal aging but can lead to mild cognitive impairment or even serious neurocognitive diseases, such as Alzheimer’s disease [26].

Age-related memory disorders include mainly dysfunctions of working memory, recent episodic memory, and learning and recall of new information, while semantic, procedural, and long-term memory are less influenced. Working memory is the coordination center of data processing, executive functions, and behavioral control in the prefrontal cortex and is connected with higher voluntary decision-making brain centers [27]. Aging affects working memory, due to decreased neuroplasticity and decreased dopaminergic and cholinergic activity in the frontal lobe, resulting in slower time-related data processing and learning difficulties [27]. The hippocampus, in particular, is a complex neuronic part of the limbic system, located in the medial temporal lobe, in functional connection with the entorhinal cortex and many other higher neuronic centers of the central nervous system [24]. Due to its complex interconnectivity, the hippocampus plays a crucial role in memory formation, memory consolidation, learning, and the integration of emotional reactions with related experiences [24]. The dentate gyrus of the hippocampus is one of the most important neuronal centers in which neurogenesis occurs during adulthood, a procedure that shows an age-related decline, apparent from early adulthood, leading to memory disturbances when it reaches a personalized threshold [24].

## Materials and methods

Objective

Schizophrenia spectrum disorders are chronic syndromes, characterized not only by a brief transient cognitive deterioration during relapse [28] but also by a gradual procedure of cognitive decline with permanent results, in addition to the consolidation of negative symptoms [9]. Age-related cognitive decline, as stated earlier, occurs gradually as a result of multiple neurodegenerative procedures in several vulnerable areas of the central nervous system and manifests itself during middle age [20]. The acknowledgment that two discrete mechanisms of cognitive decline are apparent in psychotic patients suggests a possible synergic or cumulative effect on cognitive function. Therefore, it is reasonable to question whether the anticipated age-related cognitive decline in patients diagnosed with schizophrenia spectrum disorders occurs at an accelerated rate. Furthermore, since different cognitive domains are influenced by each of the two discrete mechanisms of long-term cognitive decline, an additional aspect arises, in terms of which cognitive domains contribute mostly to the observed cognitive performance of patients diagnosed with schizophrenia spectrum disorders. In order to avoid, although, the confounding factor of transient cognitive deterioration during psychotic episodes [28], patients should be evaluated cognitively while in remission. The objective of this study is to evaluate patients diagnosed with schizophrenia spectrum disorders of different age groups, in remission, as well as healthy controls, in order to compare their performance in cognitive skills among different age groups. This comparison of cognitive abilities, while taking into account the time-dependent factor of age, will allow the evaluation of the rate of cognitive decline in psychotic patients.

Study design - participants

The study was designed as an observational, cross-sectional study. A convenience sample of patients diagnosed with schizophrenia spectrum disorders who attended the Outpatient Department of Psychosis of the University of Thessaly, Greece, and its affiliated psychiatric clinics, and a corresponding convenience sample of controls, without a diagnosis of a psychotic disorder, matched by age, gender, educational level, and ages 20-59, were selected through quota sampling and recruited from the region of Thessaly, Greece.

In order to evaluate cognitive functioning along the age spectrum, participants were divided into four age subgroups by decade, with an equal number of participants in each subgroup. The number of samples for each subgroup was calculated by power analysis, taking into consideration former studies in the general population of Greece, where the mean Montreal Cognitive Assessment (MoCA) scores for each age group, per decade, were measured [29]. The assumptions used for the calculation of the samples were an effect size of 1 and a confidence level of 95% (a=0.05). The number of samples for each subgroup was determined as 16, for theoretical power of 80% and 15, for theoretical power of 78%.

Sixty-five patients, diagnosed with schizophrenia spectrum disorders, and 64 controls, without a diagnosis of a psychotic disorder, recruited from the region of Thessaly, Greece, ages 20-59, were evaluated during the last three years, regarding their cognitive status, with the use of the Greek version of MoCA. All participants were informed about the methods and objectives of the study and written informed consent was obtained. Ethics approval was provided by the Scientific Advisory Board of the University Hospital of Larissa (8912/22-2-2018), in accordance with the 2013 revision of the Declaration of Helsinki.

The main inclusion criterion of the study for the patients is a previous diagnosis of a schizophrenia spectrum disorder. Patients should be in remission, with a maximum mean severity of psychotic symptoms of 2 (mild), as evaluated with the use of Clinician-Rated Dimensions of Psychosis Symptom Severity (CRDPSS), without a recent (less than a month) alteration of their antipsychotic medication. The inclusion criteria for the study, for the control group, are the absence of psychotic symptoms or a history of a schizophrenia spectrum disorder. The exclusion criteria for all participants included substance or medication-induced psychotic disorder, dementia, stroke, history of brain injury, epilepsy, brain tumors, encephalitis, neurodevelopmental disorders, systemic autoimmune disorders, and thyroid gland disorders. Five participants from the patient group and four participants from the control group were excluded due to the above-mentioned criteria, leaving 60 participants in the patient group and 60 participants in the control group, matched by age, gender, and educational level, divided equally into four age subgroup, per decade, with 15 participants each.

Measures

The Greek version of the MoCA scale, after officially given permission and relevant training, was administered in order to evaluate the current level of cognitive functioning of the patient and the control group, which is standardized in the Greek population, in healthy volunteers in specific diseases [29]. MoCA is a brief scale, consisting of 30 questions, on various cognitive domains, which can be assessed separately, such as visuospatial abilities and executive functioning, orientation, naming, verbal and linguistic abilities, abstract thinking, attention, and memory, especially delayed recall [30]. This scale was originally designed as a screening tool for cognitive decline in the elderly with neurocognitive disorders [30], with a cutoff point of 26/30, and is considered more sensitive than other widely used instruments. The selection of MoCA for cognitive assessment in schizophrenia spectrum disorders was based on a growing academic literature suggesting its use in recent years, due to its unique advantages for administration to psychotic patients in clinical settings, ensuring adequate cooperation, since it is brief, simple, and fast, with a mean completion time of 10 minutes [31]. The final score was adjusted to the level of education, adding one point to those with less than 12 years of formal education, according to the MoCA manual, found on the official website mocatest.org. Scores were also calculated for the cognitive domains, as presented in the MoCA scale, such as visuospatial/executive functions (0-5 points), naming (0-3 points), attention (0-6 points), language (0-3 points), abstraction (0-2 points), delayed recall (0-5 points), and orientation (0-6 points). Although it is suggested that the cut-off points for the diagnosis of cognitive decline should be adjusted for patients with schizophrenia spectrum disorders [32], it should be noted that MoCA is used in this study not as a diagnostic tool, but as a measure of cognitive performance.

CRDPSS is an eight-item measure proposed in DSM-5, which independently assesses the severity of eight important symptoms in psychotic disorders, such as the main symptoms that comprise the criteria for a categorical diagnosis of schizophrenia, such as hallucinations, delusions, disorganized speech, abnormal psychomotor behavior, and negative symptoms, together with impaired cognition, depression, and mania, in a 5-point (0-4) scale [1]. Despite being proposed for the dimensional assessment of symptoms in psychotic disorders, CRDPSS incorporates all the necessary information for the categorical diagnosis of schizophrenia spectrum disorders and specifically the presence of an active psychotic episode or remission. Therefore, all participants in the patient group were evaluated using CRDPSS, with a maximum mean severity of psychotic symptoms of 2 (mild), used as a threshold for inclusion of patients in the study.

Statistical analysis

Demographic data of all participants, such as age (both as a numerical value and as a decade-age group), educational level, and gender, as well as a categorical psychiatric diagnosis for the patient group, were recorded after being anonymously assigned a reference number, in accordance with the 2013 revision of Helsinki Declaration and the General Data Protection Regulation (GDPR). Then, participants’ current cognitive performance was assessed with the MoCA scale, and the final MoCA scores, adjusted for years of education (adding one point to the participants with less than 12 years of formal education), were used for bivariate correlation analysis with age, through the estimation of Pearson correlation coefficient. Descriptive statistics for the variables mentioned above were estimated while performing Levene’s test, Brown-Forsythe’s test, and Welch’s test, in order to evaluate the equality of variance among groups.

Moreover, the MoCA-adjusted scores, as well as all scores of each cognitive domain of the MoCA scale, such as visuospatial-executive functions, naming, attention, language, abstraction, delayed recall, and orientation, were considered as the dependent variable, while checking for differences between age subgroups, set as the independent variable, in both patients and control groups, using analysis of variance (ANOVA). The post-hoc Bonferroni test was then performed for the rejection or non-rejection of the null hypothesis, since it is considered more conservative in estimating statistically significant differences and more suitable for small samples, with few subgroups compared, along with the post-hoc Tahmane test, which does not require homogeneity of variance between groups, in case it was rejected. Finally, t-tests were performed for the comparison of mean MoCA-adjusted scores and mean cognitive domains’ scores between patients and control not only in the whole sample but also in every age subgroup separately. The level of statistical significance (a) was established at 0.05 with a confidence interval (1-a) = 5%. Statistical analyses were performed using Statistical Product and Service Solutions (SPSS, version 23; IBM SPSS Statistics for Windows, Armonk, NY).

## Results

The descriptive statistics of both patients and healthy controls are shown in Table 1. The MoCA score is negatively correlated with age, both in the patients’ group (Pearson correlation: aₚ=-0.455, p<0.001) and in the control group (Pearson correlation: aₕ=-0.404, p=0.001). The mean MoCA scores are lower in all age groups of patients diagnosed with schizophrenia spectrum disorders, compared to the same control age groups.

**Table 1:** Descriptive statistics of MoCA scores among age groups MoCA, Montreal Cognitive Assessment

		MoCA			95% Confidence Interval for Mean			
	N	Mean	Std Dev	Std Error	Lower Bound	Upper Bound	Minimum	Maximum
20-29 patients	15	26.33	1.952	0.504	25.25	27.41	23	30
30-39 patients	15	26.93	2.463	0.636	25.57	28.30	23	30
40-49 patients	16	25.50	2.191	0.548	24.33	26.67	22	30
50-59 patients	14	23.21	2.455	0.656	21.85	24.65	18	25
20-29 control	15	28.13	1.246	0.322	27.44	28.82	25	30
30-39 control	15	27.60	1.183	0.306	26.94	28.26	26	30
40-49 control	15	27.13	1.552	0.401	26.27	27.99	25	30
50-59 control	15	26.40	1.454	0.375	25.59	27.21	24	29
Total	120	26.43	2.296	0.210	26.01	26.84	18	30

While the ANOVA test was performed (p<0.001), as seen in Table 2, with the selection of the Tahmane test for post-hoc comparisons, a statistically significant difference in MoCA scores was found between the patient’s age group 50-59 and the same age group in healthy control (p=0.011). During the comparison of MoCA scores between the patients’ age groups, the oldest group 50-59 was significantly statistically differentiated from the younger groups, such as the age group 20-29 (p=0.025) and the age group 30-39 (p=0.010), except for the age group 40-49 (p=0.301). On the contrary, in healthy control age groups, such significant statistical differences were observed only between the youngest (20-29) and the oldest (50-59) age group (p=0.044), but neither the patient’s age group 50-59 and the patient’s age group 30-39 (p=0.428) nor the patient’s age group 40-49 (p=0.997).

**Table 2:** Analysis of variance (ANOVA) Note that Levene’s test p values (p<0.05) suggested the rejection of the homogeneity hypothesis in all age groups of both patients and controls. Therefore, confirmation of statistically significant differences among subgroups was based on Brown-Forsythe’s and Welch’s tests p values (p<0.05), which rejected our initial null hypothesis that such differences did not exist. Moreover, Tahmane’s test was selected for the post hoc comparisons between specific subgroups because it does not require homogeneity of variances, such as Bonferroni’s test.

ANOVA	Sum of Squares	df	Mean Square	F	Sig.
Between Groups	234.035	7	33.434	9.521	0.000
Within Groups	393.290	112	3.512		
Total	627.325	119			
MoCA	Test	Statistic	df1	df2	Sig.
Test of Homogeneity of Variances	Levene	3.222	7	112	0.003
Robust Test of Equality of Means	Welch	8.083	7	47.633	0.000
	Brown-Forsythe	9.488	7	87.658	0.000

Moreover, as seen in Table 3, while performing the t-test, there is a significant statistical difference in mean MoCA scores between patients and healthy controls, not only in the total sample (p<0.001) but also in all age groups (20-29: p=0.006; 40-49: p=0.024; 50-59: p<0.001), except for age group 30-39 (30-39: p=0.356). When t-tests were performed on the scores' specific cognitive domains, as seen in Table 4, statistically significant differences were found between patients and healthy controls in the total sample in the visuospatial and executive function domain (p=0.01), attention domain (p<0.001), language domain (p<0.001), and orientation domain (p<0.005). Regarding specific age groups, in the age group 20-29, statistically significant differences were found between patients and healthy controls in the language domain (p<0.014) and in the orientation domain (p<0.041), no difference was found in the age group 30-39, while statistically significant differences were found between patients and healthy controls in the age group 40-49 in the attention domain (p<0.001) and language domain (p<0.001). Finally, in the age group 50-59, such differences were found in the visuospatial and executive function domain (p=0.041), attention domain (p=0.006), and language domain (p=0.001). No statistically significant differences were found between patients and healthy controls in the naming domain, abstraction domain, and delayed recall domain, neither in the total sample nor in any specific age group.

**Table 3:** T-tests comparisons of MoCA scores between patients’ and control's age groups The Montreal Cognitive Assessment (MoCA) scores were adjusted for educational level, adding a point if a participant received less than 12 years of formal education. Selected p-values, depending on the acceptance of equality of variance’s assumption, are highlighted in bold. Statistically significant differences between patients and control were observed in the total sample and in all age groups, except for the age group 30-39 years old.

Age Group	MoCA - Equal Variances Assumed	Levene’s Test for Equality of Variances		T-test for Equality of Means						
				t	df	Sig. (2-tailed)	Mean difference	Std. Error Difference	95% Confidence Interval of Difference	
		F	Sig.						Lower	Upper
20-29	Yes	4.754	0.038	-3.011	28	0.005	-1.800	0.598	-3.025	-0.575
	No			-3.011	23.785	0.006	-1.800	0.598	-3.035	-0.565
30-39	Yes	10.205	0.003	-0.945	28	0.353	-0.667	0.706	-2.112	0.779
	No			-0.945	20.135	0.356	-0.667	0.706	-2.138	0.804
40-49	Yes	1.713	0.201	-2.380	29	0.024	-1.633	0.686	-3.037	-0.230
	No			-2.407	27.054	0.023	-1.633	0.679	-3.026	-0.241
50-59	Yes	5.074	0.033	-4.287	27	0.000	-3.186	0.743	-4.710	-1.661
	No			-4.214	20.832	0.000	-3.186	0.756	-4.759	-1.613
Total sample	Yes	13.575	0.000	-4.601	118	0.000	-1.783	0.388	-2.551	-1.016
	No			-4.601	93.262	0.000	-1.783	0.388	-2.553	-1.014

**Table 4:** T-test comparisons of cognitive domains’ scores between patients’ and control's age groups P-values are comparatively and concisely displayed, while statistically significant differences are highlighted in bold. Note that different cognitive domains contribute to MoCA differences, in each age subgroup. Nevertheless, naming, abstract thinking, and delayed recall’s differences between patients and control were not statistically significant either in any age group or the total sample.

Age Group	T-test for Equality of Means (p values)							
	Visuospatial/Executive functions	Naming	Attention	Language	Abstraction	Delayed recall	Orientation	MoCA
20-29	0.109	1	0.075	0.014	0.638	0.812	0.041	0.006
30-39	0.616	1	0.410	0.711	0.164	0.863	0.559	0.356
40-49	0.330	1	0.013	0.017	0.930	0.937	0.164	0.024
50-59	0.041	0.336	0.006	0.001	0.647	0.751	0.165	0.000
Total sample	0.010	0.651	0.000	0.000	0.695	0.865	0.005	0.000

## Discussion

The results of our research contribute to the academic literature by supporting the assumption that cognitive decline in patients diagnosed with schizophrenia spectrum disorders occurs earlier, compared to the general population, with the novel finding that this cognitive decline occurs at an accelerated rate after middle age. Schizophrenia spectrum disorders have a negative impact on cognitive performance, as expected, since the patient’s group, compared to the control group, had statistically significant lower MoCA scores in most age groups, as well as in the entire sample. It should be noted that the descriptive statistics of our control group’s cognitive performance were similar to those of larger multicenter studies that provide normative data on the performance of the MoCA scale in the Greek population [29].

Nevertheless, an additional novel finding is that our results indicate that different cognitive domains contribute to the observed cognitive decline between patients and controls in each age group, suggesting an age-specific pattern of decline in cognitive domains in different age groups. Specifically, in the total sample, patients had statistically significant differences from controls in visuospatial and executive functions, attention, linguistic skills, and orientation. However, these cognitive domains did not differentiate cognitively between patients and control in all age groups, except for linguistic skills, such as repetition and verbal flow. In the youngest age group (20-29 years old), only linguistic skills and orientation were lower, while in the older age groups, lower patient scores were detected in the attention and linguistic skills in 40-49 years old age group, with the addition of visuospatial and executive functions in 50-59 years old age group. This result could be attributed to the theoretical procedure of increased frontoparietal cognitive effort observed in psychotic patients during the early stages, which decompensates after a specific threshold is reached [17], offering also a possible order of cognitive domain decompensation.

Furthermore, age was negatively correlated with cognitive performance, both between the patient and the control group, suggesting that age is indeed a second factor that deteriorates cognition. An interesting finding is that some cognitive domains, such as naming, abstraction, and delayed recall, did not contribute to distinguishing patients from controls in any age group, suggesting that they are not influenced by psychosis-related cognitive procedures. Age-related neurodegenerative procedures seem to affect only these cognitive domains, a finding that is particularly obvious, especially for delayed recall, and is supported by the current academic literature [24,26].

Our research indicates that cognitive decline occurs at an accelerated rate after middle age. This notion is based on several findings from our research. At first, a notable exception was found in the patient's 30-39 age group, where cognitive performance was lower than in the respective control group, but the difference was not statistically significant. Moreover, it was slightly higher (26.93 vs. 26.4) than the younger patient’s age group 20-29. This exception can differentiate the estimated curve of cognitive performance with age, for the patient group and control group. Specifically, while in the control group, there is a clear linear decline in MoCA scores with age, in the patient group cognitive performance rises at first, reaching a maximum value and then declines with an accelerated rate, compared to the linear decline seen in the control group, where only age affects cognitive performance.

These findings are consistent with other research results, since an initial increase in cognitive deterioration in psychotic patients is seen in academic literature, not only during the prodromal phase and in relation to the duration of untreated psychosis [28], but also during the first psychotic episode, as seen in a 6-year follow-up study [9], where the initiation of antipsychotic medication and maintenance of remission was followed by improvements in many cognitive domains. Given that the age group 20-29 is the mean period in which psychotic disorders are diagnosed, especially in men [10], during which antipsychotic medication may not be administered [19] or psychotic episodes can be undertreated, due to initial diagnostic uncertainty, cognitive improvement, at least in the short term, along with remission of other psychotic symptoms, is reasonable to be expected after the initiation of antipsychotic medication. In the long term, however, metabolic side effects, especially of widely used second-generation antipsychotics, can be partially responsible for the observed accelerated rate of cognitive decline [19], through vascular damage of the central nervous system. Further multicenter research should focus on clarifying the validity of these assumptions.

An additional finding supporting the accelerated rate of cognitive decline in psychotic patients is that the oldest patients’ age group (50-59) had a significant difference in cognitive performance not only from the youngest age group (20-29), as found in the control group, but also from the 30-39 age group. Moreover, such a statistically significant difference is not observed between patients’ age groups 20-29 and 40-49, which have the same age difference of 20 years as the groups 30-39 and 50-59. Therefore, these findings could be interpreted as indicating that, in the second two-decade period, the decline rate is faster. Although a possible confounding factor could be the lower educational level of older participants, it should be noted that cognitive performance was evaluated using the adjusted-for-educational level MoCA score.

Finally, the finding that different cognitive domains are influenced in the psychotic patients’ age subgroups further supports the assumption that cognitive decline accelerates as age increases. Specifically, after middle age, namely, after 40 years old, linguistic skills and attention initially seem to be more distorted in patients diagnosed with a schizophrenia spectrum disorder than in the control group. In the next decade age group (50-59 years old) an additional cognitive domain, meaning visuospatial and executive functions, is also influenced. This finding suggests that, since more cognitive domains seem to decompensate after the age of 50 years old, cognitive decline appears to be faster than in younger age groups.

Limitations

The main limitations of our study, in an attempt to draw general conclusions from our findings, are the limited geographical range of sample selection, which was restricted to the region of Thessaly in Central Greece, the brief assessment of cognitive performance, without the use of other larger cognitive assessment batteries, specific for schizophrenia and the size of our sample, which was relatively small. The existence of fewer participants per age group would reduce the statistical power of our research findings to less than 78%, especially since the oldest patients’ age group of 50-59 years was slightly smaller than the other age groups. This may be attributed to the selection method for our sample, which resulted in the underrepresentation of older patients diagnosed with schizophrenia spectrum disorders. Research in tertiary clinical centers is often susceptible to Berkson’s bias, which is observed when the sample is limited in help-seeking populations and is proportional to the level of specialization of the research center.

## Conclusions

Subsequently, despite the limitations of our study, our results are similar to other studies suggesting that deterioration in cognitive performance in patients diagnosed with schizophrenia spectrum disorders occurs faster than in the general population, as a result of the cumulative chronic effect of age and psychosis. Furthermore, our results propose the novel finding that this occurs at an accelerated rate in relation to the control sample, which is more evident as age increases, while specific cognitive domains’ decline patterns are found in different age groups. Therefore, based on the observation of apparent cognitive decline in psychotic patients, regular evaluation of cognitive functioning in schizophrenia spectrum disorders in clinical settings is highly suggested. Moreover, the use of measures suitable for the dimensional approach of psychosis, such as CRDPSS, allows correlations between psychotic symptoms and especially between acute cognitive decline and psychotic relapse, presents specific advantages while evaluating cognition. Future research focusing on larger, multicenter studies should further highlight the cumulative effects of psychosis and aging in cognitive domains, allowing its scientific manifestation and implementation of new schizophrenia-specific cognitive assessment scales, as well as evidence-based procognitive intervention strategies, not only medical but also through psychotherapy, psychoeducation, neuropsychological, cognitive, and metacognitive training, as part of their holistic treatment.

## References

[1] American Psychiatric Association (2013). Diagnostic and Statistical Manual of Mental Disorders, Fifth Edition.

[2] Tandon R, Gaebel W, Barch DM (2013). Definition and description of schizophrenia in the DSM-5. Schizophr Res.

[3] Guloksuz S, van Os J (2018). The slow death of the concept of schizophrenia and the painful birth of the psychosis spectrum. Psychol Med.

[4] Hochberger WC, Thomas ML, Joshi YB (2020). Deviation from expected cognitive ability is a core cognitive feature of schizophrenia related to neurophysiologic, clinical and psychosocial functioning. Schizophr Res.

[5] Seidman LJ, Mirsky AF (2017). Evolving notions of schizophrenia as a developmental neurocognitive disorder. J Int Neuropsychol Soc.

[6] Palaniyappan L, Hodgson O, Balain V, Iwabuchi S, Gowland P, Liddle P (2019). Structural covariance and cortical reorganisation in schizophrenia: a MRI-based morphometric study. Psychol Med.

[7] Zhu J, Qian Y, Zhang B, Li X, Bai Y, Li X, Yu Y (2020). Abnormal synchronization of functional and structural networks in schizophrenia. Brain Imaging Behav.

[8] Besteher B, Brambilla P, Nenadić I (2020). Twin studies of brain structure and cognition in schizophrenia. Neurosci Biobehav Rev.

[9] Fu S, Czajkowski N, Torgalsbøen AK (2018). Cognitive improvement in first-episode schizophrenia and healthy controls: a 6-year multi-assessment follow-up study. Psychiatry Res.

[10] Sheffield JM, Karcher NR, Barch DM (2018). Cognitive deficits in psychotic disorders: a lifespan perspective. Neuropsychol Rev.

[11] Schizophrenia Working Group of the Psychiatric Genomics Consortium (2014). Biological insights from 108 schizophrenia-associated genetic loci. Nature.

[12] Mark W, Toulopoulou T (2016). Cognitive intermediate phenotype and genetic risk for psychosis. Curr Opin Neurobiol.

[13] Ross RM, McKay R, Coltheart M, Langdon R (2016). Perception, cognition, and delusion. Behav Brain Sci.

[14] Bagney A, Rodriguez-Jimenez R, Martinez-Gras I (2013). Negative symptoms and executive function in schizophrenia: does their relationship change with illness duration?. Psychopathology.

[15] Livingston G, Huntley J, Sommerlad A (2020). Dementia prevention, intervention, and care: 2020 report of the Lancet Commission. Lancet.

[16] Mihaljević-Peleš A, Bajs Janović M, Šagud M, Živković M, Janović Š, Jevtović S (2019). Cognitive deficit in schizophrenia: an overview. Psychiatr Danub.

[17] Brandt CL, Kaufmann T, Agartz I (2015). Cognitive effort and schizophrenia modulate large-scale functional brain connectivity. Schizophr Bull.

[18] Na KS, Jung HY, Kim YK (2014). The role of pro-inflammatory cytokines in the neuroinflammation and neurogenesis of schizophrenia. Prog Neuropsychopharmacol Biol Psychiatry.

[19] MacKenzie NE, Kowalchuk C, Agarwal SM (2018). Antipsychotics, metabolic adverse effects, and cognitive function in schizophrenia. Front Psychiatry.

[20] da Costa JP, Vitorino R, Silva GM, Vogel C, Duarte AC, Rocha-Santos T (2016). A synopsis on aging-theories, mechanisms and future prospects. Ageing Res Rev.

[21] Kubben N, Misteli T (2017). Shared molecular and cellular mechanisms of premature ageing and ageing-associated diseases. Nat Rev Mol Cell Biol.

[22] Ungvari Z, Tarantini S, Donato AJ, Galvan V, Csiszar A (2018). Mechanisms of vascular aging. Circ Res.

[23] Satoh A, Imai SI, Guarente L (2017). The brain, sirtuins, and ageing. Nat Rev Neurosci.

[24] Hullinger R, Puglielli L (2017). Molecular and cellular aspects of age-related cognitive decline and Alzheimer's disease. Behav Brain Res.

[25] Huang Z, Jordan JD, Zhang Q (2023). Early life adversity as a risk factor for cognitive impairment and Alzheimer's disease. Transl Neurodegener.

[26] Fjell AM, Sneve MH, Grydeland H, Storsve AB, Walhovd KB (2017). The disconnected brain and executive function decline in aging. Cereb Cortex.

[27] Froudist-Walsh S, López-Barroso D, José Torres-Prioris M, Croxson PL, Berthier ML (2018). Plasticity in the working memory system: life span changes and response to injury. Neuroscientist.

[28] Bora E, Yalincetin B, Akdede BB, Alptekin K (2018). Duration of untreated psychosis and neurocognition in first-episode psychosis: a meta-analysis. Schizophr Res.

[29] Konstantopoulos K, Vogazianos P, Doskas T (2016). Normative data of the Montreal cognitive assessment in the Greek population and Parkinsonian dementia. Arch Clin Neuropsychol.

[30] Nasreddine ZS, Phillips NA, Bédirian V (2005). The Montreal cognitive assessment, MOCA: a brief screening tool for mild cognitive impairment. J Am Geriatr Soc.

[31] Yang Z, Abdul Rashid NA, Quek YF (2018). Montreal cognitive assessment as a screening instrument for cognitive impairments in schizophrenia. Schizophr Res.

[32] Gil-Berrozpe GJ, Sánchez-Torres AM, García de Jalón E, Moreno-Izco L, Fañanás L, Peralta V, Cuesta MJ (2020). Utility of the MoCA for cognitive impairment screening in long-term psychosis patients. Schizophr Res.

